# Effect of climate change, extreme temperatures (heat and cold) on diabetes mellitus risk, hospitalization, and mortality: Global Evidenced Based Study

**DOI:** 10.3389/fpubh.2025.1677522

**Published:** 2025-10-16

**Authors:** Sultan Ayoub Meo, Narmeen Shaikh, Farah Adnan Abukhalaf, Anusha Sultan Meo, David C. Klonoff

**Affiliations:** ^1^Department of Physiology, College of Medicine, King Saud University, Riyadh, Saudi Arabia; ^2^College of Medicine, King Saud University, Riyadh, Saudi Arabia; ^3^The School of Medicine, Medical Sciences and Nutrition, University of Aberdeen, Aberdeen, United Kingdom; ^4^Department of Clinical Medicine, University of California, San Francisco, San Francisco, CA, United States; ^5^Mills-Peninsula Medical Center, San Mateo, CA, United States

**Keywords:** climate change, extreme temperatures, heat, cold, diabetes mellitus

## Abstract

**Background:**

Climate change and diabetes mellitus are challenging threats to humanity in the 21st century. Climate change enhances the occurrence and severity of extreme temperature events, heat and cold, which can lead to severe health consequences. This study aimed to investigate the effects of extreme temperatures, including heat and cold, on the risk of developing diabetes mellitus, requiring hospitalizations or emergency department (ED) visits, and deaths.

**Methods:**

In this study, 116 documents were initially identified from “databases, including PubMed, Web of Science, Scopus, and Google Scholar.” Articles on extreme temperatures, heat, cold, and diabetes mellitus were searched using the keywords: climate change, extreme temperatures, heat, cold, and diabetes mellitus. The descriptive information was recorded from the identified studies. Eventually, 13 documents were included in the analysis and synthesis. The association between extreme temperatures, including heat and cold, and diabetes-related outcomes, such as diabetes risk, hospitalizations, ED visits, and mortality was established.

**Results:**

Exposure to extreme temperatures (heat and cold) were positively and significantly related with an increased risk of adverse diabetes-related events, with a combined risk ratio (RR) of 1.14 (95% CI: 1.08–1.21, *p* = 0.01); diabetes-related hospitalizations and emergency department (ED) visits (RR of 1.10, 95% CI: 1.01–1.19, *p* = 0.03); and increased diabetes-linked mortality (RR 1.16, 95% CI: 1.07–1.25, *p* = 0.01).

**Conclusions:**

Extreme temperatures (heat and cold) significantly increased adverse diabetes-related events, hospitalizations, emergency department visits, and diabetes-related mortality. Reducing the risk of climate change and extreme temperatures requires coordinated efforts at individual, community, national and global levels to combat climate change and diabetes mellitus.

## 1 Introduction

Diabetes mellitus (DM) is a global primary public health concern that can lead to numerous health problems. Worldwide, approximately 589 million people have diabetes, which represents 11.1% of the world population, resulting in 3.4 million diabetes-related deaths annually, with a USD 1 trillion loss in health expenditure in 2024. The number of diabetes cases is anticipated to reach 852.5 million by 2050 (1). The incidence of diabetes has increased because of a sedentary lifestyle, unhealthy diet, and unfavorable living environments (1). In addition to these factors, climate change and extreme temperatures have emerged as significant risk factors for DM (2).

DM and climate change are two distinct but interconnected conditions that pose significant risks to human health. People with DM face increased threats from climate change, including extreme heat, cold, and natural disasters (3). Extreme weather events and rising temperatures lead to higher morbidity and mortality among individuals with DM (4). DM admissions due to extreme cold exposure were higher during the winter months. Women, older adults, urban residents, and those with multiple comorbidities are more vulnerable to extreme weather events (5).

Human activities, urbanization, industrialization, and the emission of greenhouse gases (GHGs) contribute to global warming, which causes extreme heat. Climate change causes disproportionately adverse effects on marginalized and vulnerable populations. Climate change impacts human health through both direct pathways, such as extreme heat, cold, drought, flooding, and wildfires, as well as indirect pathways involving the ecosystem, food and nutrition security, water quality, disease vector distribution, and economies (6).

The effects of climate change are felt across regions, genders, income levels, social classes, ethnic backgrounds, ages, and physical abilities. The severity, nature, and timing of adverse health impacts are complex and vary within regions; however, there are challenges for an equitable climate-health transition that can be addressed through more integrated mitigation and adaptation strategies. The climate crisis is not only an environmental emergency but also a growing public health challenge. In 2022 and 2023, over 100,000 people across 35 countries in the European Region died due to heat-related causes (7). The report predicts that record-breaking extreme temperatures are becoming frequent, intense, and prolonged because of human-induced climate change (8). Extreme temperatures cause about 5 million deaths each year worldwide (9), account for 13% of cardiovascular deaths (10) and 5.2% of stroke deaths globally (11).

Climate change poses significant health risks, resulting in humanitarian emergencies triggered by heatwaves, wildfires, floods, storms, and hurricanes. Nearly 3.6 billion people are living in areas which are highly vulnerable to climate change. It is estimated that, in the future, climate change could result in 250,000 additional deaths annually (12). The primary impact of climate change, including extreme temperatures (both hot and cold), creates significant global public health challenges. There is a lack of research on how extreme temperatures specifically affect diabetes mellitus. Thus, this study aims to explore the impact of hot and cold temperatures on the risk of diabetes mellitus, hospitalization, emergency department (ED) visits, and mortality.

## 2 Research methodology

### 2.1 Study design and settings

This study was conducted in the “Department of Physiology, College of Medicine, King Saud University, Riyadh, Saudi Arabia” from January 2025 to May 2025.

### 2.2 Selection of studies

**Figure 1:**
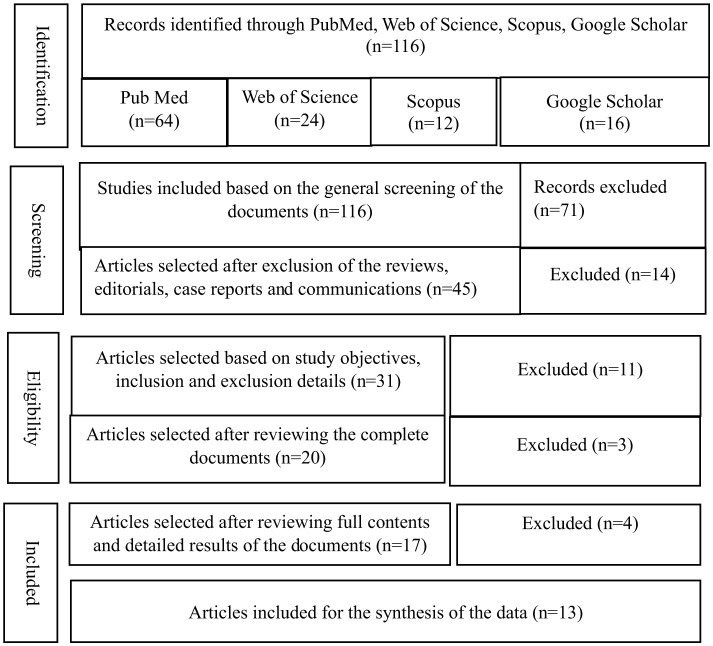
PRISMA: for the selection of documents.

A wide-ranging search was performed to identify the literature on the impact of extreme temperatures, including heat and cold, on the risk of diabetes mellitus, hospitalization, emergency department visits, and mortality. The literature was searched across “PubMed, Web of Science, Scopus, and Google Scholar.” The search terms employed were “extreme temperatures, weather conditions, climate change, heat, cold, diabetes mellitus, prevalence, risk, hospitalization, and mortality.” Initially, 116 documents were identified; after reviewing the abstract and detailed article, 13 articles were included (Table 1, Figure 1). A PRISMA flow diagram was used to document the selection process for relevant articles and documents that demonstrate the impact of extreme temperatures, heat and cold on diabetes mellitus (Figure 1).

**Table 1:** Impact of extreme temperatures (heat and cold) on the risk of diabetes mellitus, hospitalization, emergency department visits, and mortality.

**Author, Year of Study, Country**	**Study type**	**Sample size**	**Outcome**
Xu et al., 2021, Brazil (34)	Case-crossover	553,351 hospitalizations	5 °C rise in day-to-day mean temperature (lag 0–3 days) causes an increase in OR = 1.06; 95% CI: 1.04–1.07
Kim et al., 2022, South Korea (35)	Nationwide multi-region time-series	Data from 16 regions	Hospital admission: (RR = 1.45; 95% CI: 1.26–1.66). Mortality due to diabetes was linked with exposure to cold spells: (RR = 2.02; 95% CI: 1.37–2.99)
Tao et al., 2023, China (36)	Case-crossover analysis	18,685 diabetes deaths	• Heatwaves increase diabetes mortality. • Rural: OR = 1.19 (95% CI: 1.14–1.25) • Urban: OR = 1.09 (95% CI: 1.05–1.12)
Xu et al., 2019, Australia (23)	Population-based retrospective cohort study	10,542 hospitalizations for DM; 513 post-discharge deaths due to DM	Heatwaves cause a significant impact on hospital admissions for diabetic patients (OR: 1.07; 95% CI: 1.01, 1.15; *p* = 0.039) and deaths due to diabetes (OR: 1.68; 95% CI: 1.10, 2.58; *p* = 0.017)
Wang et al., 2024, UK (37)	Prospective cohort study	103,215 participants	Compared to a comfortable workplace temperature, the risk of type 2 diabetes increased in cold environments (HR = 1.27, 95% CI: 1.04–1.55) and hot environments (HR = 1.32, 95% CI: 1.17–1.48)
Li et al., 2014, China (16)	Time-series study (DLNM)	• Harbin: 4.7 million Chongqing: 1.8 million • Total: 6.5 million	• In Harbin, a 1 °C increase in temperature from 29 °C (heat) was associated with a high diabetes mortality risk, with an RR of 1.165 (95% CI 1.038, 1.391), and a 1 °C decrease from 11 °C (cold) was linked to an RR of 1.129 (95% CI 1.025, 1.337), respectively • In Chongqing, a 1 °C increase (heat) from 29 °C and a 1 °C decrease (cold) from −11 °C were linked with a 1.197 (1.039, 1.485) and 1.125 (0.953, 1.475) increase in diabetes mortality risk, respectively
Bai et al., 2016, Canada (38)	Time-series study	324,034 diabetes hospital admissions	Diabetes related hospitalizations increased by 12% (RR = 1.12; 95% CI: 1.01–1.24) with cold temperatures (1st percentile vs MMP) and by 30% (RR = 1.30; 95% CI: 1.06–1.58) with hot temperatures (99th percentile vs. MMP)
Isaksen et al., 2016, USA (39)	Time-series analysis;	135,333 deaths	RR (95% CI) for mortality on a 99th percentile (36.1 °C) heat day compared with a non-heat day for all ages for DM was 1.2 (0.93, 1.55)
Zanobetti et al., 2012, USA (40)	Time series	• 135 US cities • 3,364,868 with diabetes	1 °C rise in summer temperature change (SD) was linked to high mortality risk in people with diabetes (HR = 1.040; 95% CI: 1.022–1.059).
Hajat et al., 2017, England (41)	Case-crossover study	4,474,943 consultations	Rise in temperature above 22 °C (heat effect) OR = 1.097 (95% CI: 1.041–1.156). For temperatures below 0 °C (cold effect) OR = 1.024 (95% CI: 1.019–1.030)
Ma et al., 2020, China (42)	Multicity time-series	1,368,648 cases of death	Diabetes mortality was significantly linked to extreme heat RR, 1.35; 95% CI, 1.08–1.68 (97.5th vs. MMP) but not with cold effect with RR: 1.24; 95% CI, 0.84, 1.82 (2.5th vs. MMP)
Knowlton et al., 2009, USA (43)	Time series	501,951 ED visits during a heat wave, 485,785 visits in the non-heatwave	Heat wave caused hospitalization for diabetic patients (RR: 1.01, 95% CI 0.99–1.03)
Winquist et al., 2016, USA (44)	Time series	9,856,015 ED visits	Significant associations were observed at lag 0 for all ages combined, with a relative risk RR 1.034 (95% CI, 1.014–1.054) for ED visits, temperature change of 27–32 °C

### 2.3 Exposure definition

In this study, we compiled data from various studies where researchers used different exposure definitions: daily mean temperature during the hottest and coldest months; daily mean temperature during the cold season (Nov–Mar); during the warm season (May–Sep); and temperature changes of 1 °C increase or decrease. Extreme cold was defined by the 1st and 5th percentiles of daily maximum temperature, while extreme heat was defined by the 95th and 99th percentiles, and daily maximum temperature was also considered. Daily mean temperature thresholds were set at heat = per 1 °C above 22 °C, cold = per 1 °C below 0 °C; and extreme heat at the 97.5th percentile vs. extreme cold at the 2.5th percentile. Additionally, heatwaves were characterized as periods of ≥2 consecutive days with temperatures at or above the 90th−99th percentile of daily mean temperature.

### 2.4 Statistical analysis

This analysis included studies that reported associations between heat or cold exposure and diabetes outcomes, using “risk ratios (RR), hazard ratios (HRs), or odds ratios (ORs)” as effect measures. We did not calculate these effect estimates ourselves; they were extracted as reported from the original studies. To enable a consistent analytic synthesis, all effect estimates were converted to risk ratios (RRs) where appropriate. For studies reporting hazard ratios, no conversions were applied. This is because the event rates were low across these studies, making HRs a reasonable approximation of RRs without the need for transformation (13). For studies reporting odds ratios, if the baseline event rate was low ( ≤ 10%), then the OR was treated as an approximate RR (14). For studies using case-crossover designs, such as those reporting associations between heat exposure and diabetes-related hospitalizations, we treated odds ratios (ORs) as approximations of relative risks (RRs). This was justified by the low daily probability of hospitalization events, which satisfies the rare outcome assumption under which OR ≈ RR.

All effect estimates were interpreted in line with their original definitions. The definitions of “extreme cold” and “extreme heat” were not standardized across the studies. Instead, we preserved each study's own definition of temperature extremes, as these were determined based on local climatic conditions or temperature percentiles relevant to each study's geographic and contextual setting. Meta-analyses were conducted only when three or more studies were available for a given exposure-outcome group. The primary analysis included all eligible studies that reported incident diabetes, hospitalizations, emergency department visits, or mortality to evaluate the overall relationship between extreme weather and diabetes burden. Subgroup analyses were conducted for: (a) hospitalization/ED visits, and (b) mortality. Incidence was not included in the subgroup analysis because it was based on only one contributing study.

When a single study reported effect estimates for both heat and cold effects, each extreme temperature was used as a separate data point, when a single study also reported distinct subpopulations (e.g., rural vs. urban regions), each subgroup was treated as a separate data point in the meta-analysis, provided the estimates were independent and based on non-overlapping populations.

The analyses were performed using “RStudio version 4.3.2 and the package ‘meta'.” The heterogeneity among the “pooled studies was evaluated using the Cochrane chi-square test (*Q*) and *I*^2^. Moderate to a high degree of heterogeneity was indicated when the *p*-value of the chi-square test was < 0.05 and the *I*^2^ value was ≥50%, and in that case, the random effect model” was applied by established guidelines (15). The overall effect “estimate was considered statistically significant when the *p*-value was < 0.05. Publication bias was evaluated using Egger's regression test” and visual inspection of funnel plots. To assess the robustness of the results, a leave-one-out sensitivity analysis was performed by sequentially omitting each study to determine its impact on the pooled effect estimates.

## 3 Results

**Figure 2:**
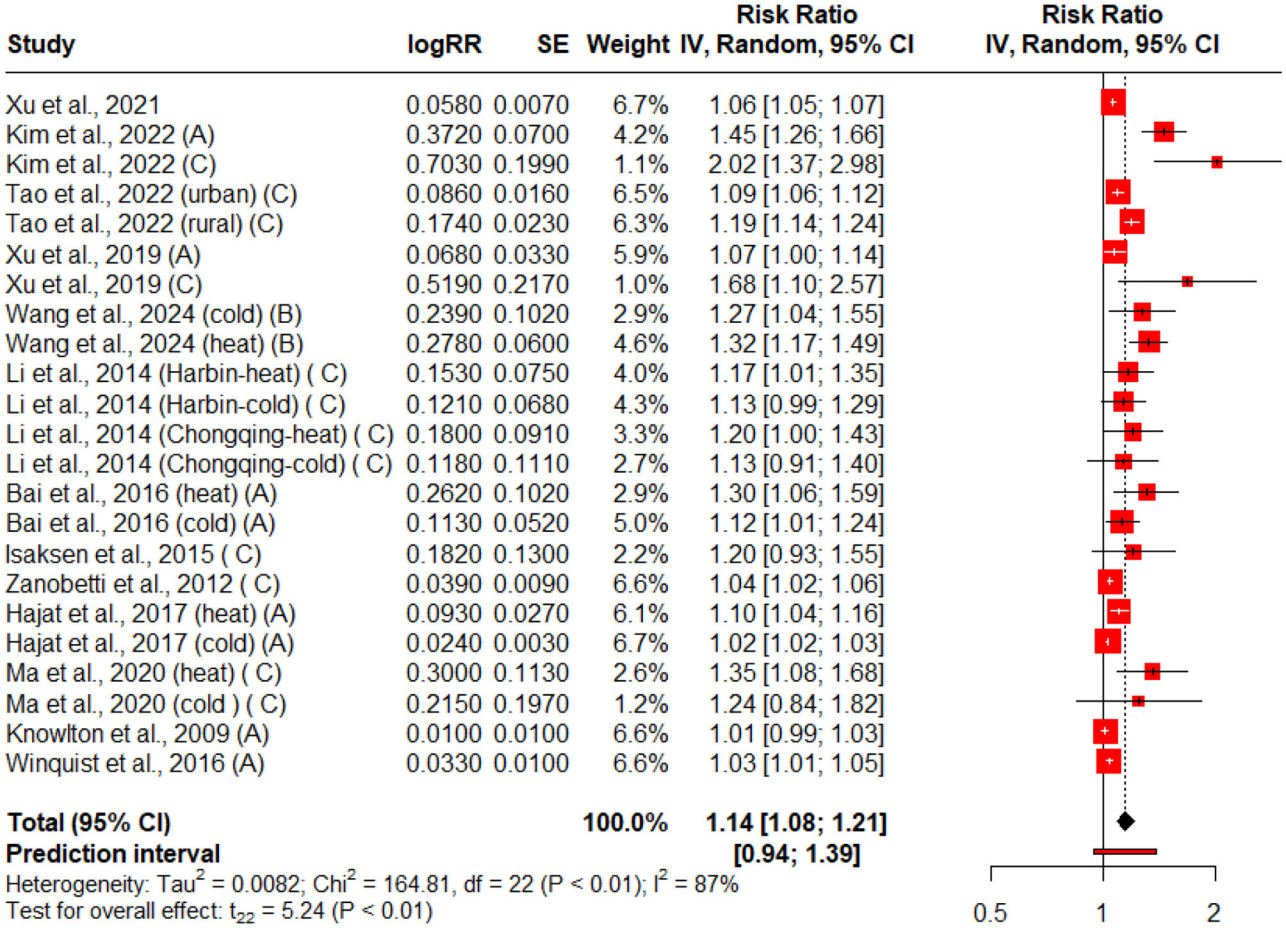
Forest plot for the association of adverse diabetes events with extreme temperature exposure. The black diamond represents the combined RR for all the studies. The red squares represent the individual RR for each survey. The solid vertical line represents RR = 1. The dashed line represents the point estimate of the overall RR for all studies. A, B, and C represent different adverse diabetes events: A (hospitalization/ED visits), B (incidence), and C (mortality).

**Figure 3:**
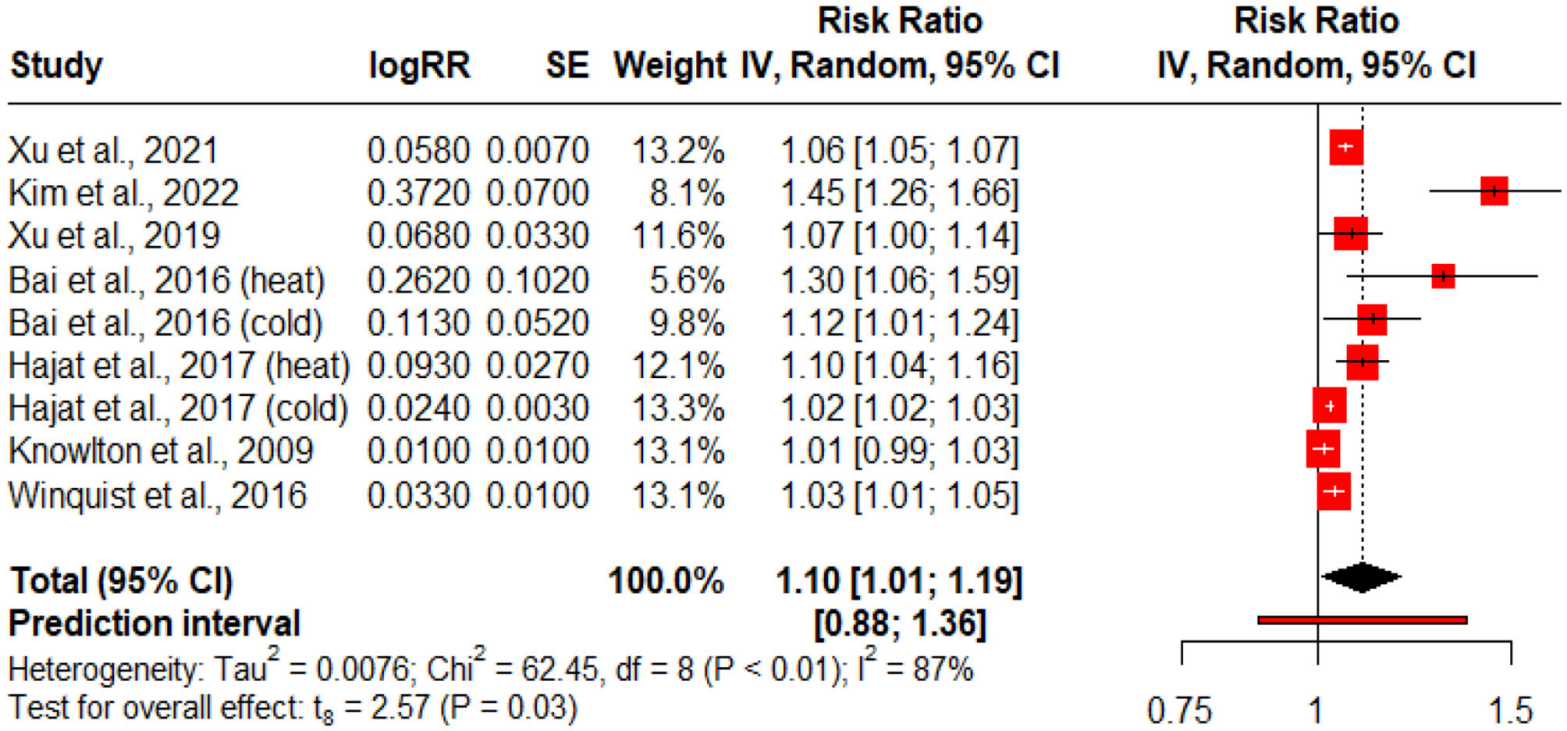
Forest plot for the association of diabetes hospitalization/ED visits with extreme temperature exposure. The black diamond represents the combined RR for all the studies. The red squares represent the individual RR for each study. The solid vertical line represents RR = 1. The dashed line represents the point estimate of the overall RR for all studies.

Table 1 illustrates the impact of extreme temperatures (both heat and cold) on the risk of diabetes mellitus, hospitalization, emergency department visits, and mortality. A total of thirteen studies were included (Table 1). Forest plot for the association between extreme temperatures and adverse diabetes events (Figure 2), hospitalization, emergency department visits (Figure 3), and diabetes allied mortality (Figure 3) were highlighted. However, we were unable to present a forest plot for the incidence of diabetes correlated with extreme temperature. The reason is that there were not enough incidence studies for a robust forest plot.

### 3.1 Extreme temperature exposure and all diabetes outcomes

Figure 2 presents a forest plot summarizing the association between extreme weather exposure (heat and cold) and all diabetes-related consequences, including hospitalizations, emergency department (ED) visits, mortality, and incidence rates. Some studies contributed multiple data points when they reported on different exposure types (e.g., heat vs. cold) or population subgroups (e.g., urban vs. rural). Because of significant between-study heterogeneity (*Q* = 164.81, *p* < 0.01; *I*^2^ = 87%), a random-effects model was applied. The pooled analysis revealed a statistically significant positive association, with a combined risk ratio (RR) of 1.14 (95% CI: 1.08–1.21, *p* < 0.01), indicating that extreme temperatures are linked to an increased risk of adverse diabetes-related events.

### 3.2 Extreme temperature exposure and diabetes related hospitalization/ED visits

Figure 3 displays a forest plot summarizing the association between extreme temperature exposure (heat and cold) and diabetes-related hospitalizations and emergency department (ED) visits. This subgroup analysis includes studies that specifically measured outcomes related to acute healthcare utilization. Where studies reported multiple subgroups (e.g., heat vs. cold), each was included as a separate data point to preserve exposure specificity. A random-effects model was used due to substantial between-study heterogeneity (*Q* = 62.45, *p* < 0.01; *I*^2^ = 87%). The pooled estimate indicated a statistically significant positive association, with a combined risk ratio (RR) of 1.10 (95% CI: 1.01–1.19, *p* = 0.03).

### 3.3 Extreme temperature exposure and diabetes related mortality risk

**Figure 4:**
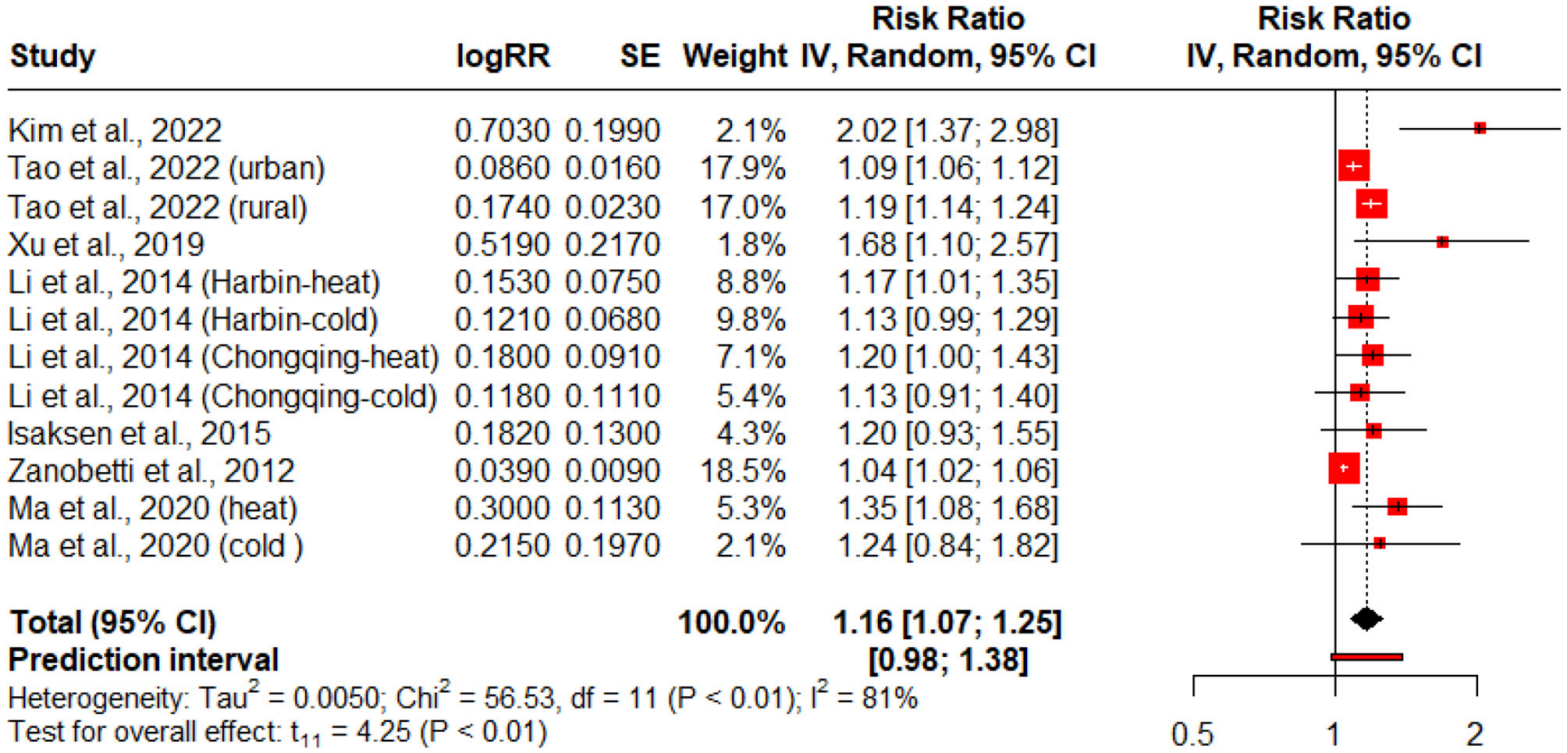
Forest plot for the association of diabetes mortality risk with extreme temperature exposure. The black diamond represents the combined RR for all the studies. The red squares represent the individual RR for each study. The solid vertical line represents RR = 1. The dashed line represents the point estimate of the overall RR for all studies.

Figure 4 presents the forest plot summarizing the association between exposure to extreme temperatures (heat and cold) and diabetes-related mortality. This subgroup analysis includes studies that specifically reported mortality outcomes. Where studies provided distinct estimates for different temperature exposures (e.g., heat vs. cold) or population subgroups, each was included as a separate data point to ensure exposure-specific precision. Due to moderate heterogeneity across studies (*Q* = 56.53, *p* < 0.01; *I*^2^ = 81%), a random-effects model was used. The pooled risk ratio (RR) was 1.16 (95% CI: 1.07–1.25, *p* < 0.01), indicating a significant relationship between extreme temperature exposure and increased risk of diabetes-related deaths.

## 4 Discussion

Climate change poses a primary threat to the entire environment, including both natural and human systems, as well as human health, social, and economic conditions. Climate change exacerbates the frequency and severity of heat and cold events, and an increase in high temperatures can lead to severe health consequences. The results of this study revealed that extreme temperatures significantly increase the adverse diabetes-related events, hospitalizations, (ED visits, and diabetes-related mortality; Table 1, Figures 2–4). Extreme temperatures are increasingly recognized as critical environmental stressors that significantly impact individuals with diabetes mellitus (DM). Patients with diabetes are vulnerable to impaired thermoregulation, autonomic dysfunction, and possible coexisting complications (16). Extreme temperatures have been linked to diabetes-associated adverse events, but the limited literature has explored this association in depth.

People with diabetes mellitus are highly vulnerable during extreme heat and cold events (17, 18). In contrast, the medical literature suggests that individuals with diabetes are potentially more susceptible to the effects of cold stress (19). The literature in this area highlights the possible consequences of cold exposure on people with type 2 diabetes (20, 21).

He et al. (22) explored the link between heat and diabetes mortality and reported that heat exposure is a risk factor for death among the diabetic population. Additionally, they found that individuals with diabetes residing in areas of high greenness have a lower risk of heat-related mortality.

Li et al. (16) reported that both extreme cold and heat had a significant association with “diabetes mortality. The extreme heat effects were acute and short-term; however, the extreme cold effects were delayed and long-term.” Similarly, Xu et al. (23) assessed the impact of heatwaves on hospitalizations and deaths among individuals with diabetes. The authors found a significant rise in mortality. During a middle-intensity 2-day heat wave, hospitalizations for diabetes increased by 19% and deaths increased by 64%. However, during a high-intensity heat wave for 2 days, hospitalization for diabetes increased by 37% and fatalities increased by 137%. The most vulnerable populations for heatwaves were children and males with diabetes. The authors concluded that heat wave was the leading cause of hospitalizations and premature deaths among people with diabetes.

Gao et al. (24) demonstrated that extreme heat exposure caused a rising incidence of diabetes-related hospitalizations. In another study, Song et al. (25) found that both heat and cold had an impact on diabetes-related morbidity and mortality. The results further revealed that the effect of heat exposure on diabetes mortality was greater than the effect on morbidity. The authors concluded that heat and cold temperatures caused diabetes mortality.

Blauw et al. (26) reported that global rising temperatures play a role in the current diabetes epidemic situation worldwide. The higher ambient temperatures can negatively affect glucose metabolism by reducing the function of brown adipose tissue. The authors demonstrated that, on average, a 1 °C rise in temperature was associated with an increase in diabetes incidence. They reported that the prevalence of glucose intolerance increased following as little as a 1 °C rise in temperature. They also reported that a 1 °C rise in temperature accounts for 100,000 new cases of diabetes per annum in the USA. Moon (27) demonstrated that the pooled risk of increased mortality and morbidity for people with diabetes during a heat wave was 18% for mortality and 10% for morbidity. Yang et al. (28) examined the impact of ambient temperatures on diabetes mortality in China. The authors found that the heat effect was generally acute and followed by mortality. The relative risks of extreme heat effects were higher in females than in males. The impacts of heat and cold were greater among the older population and those with lower levels of education. Thus, most studies in the literature are in agreement with the findings in this article that extreme temperatures (heat and cold) significantly increase the incidence of adverse diabetes-related events, hospitalizations, emergency department (ED) visits, and diabetes-related mortality.

### 4.1 Pathophysiology: extreme temperatures, DM risk, hospitalization and mortality

**Figure 5:**
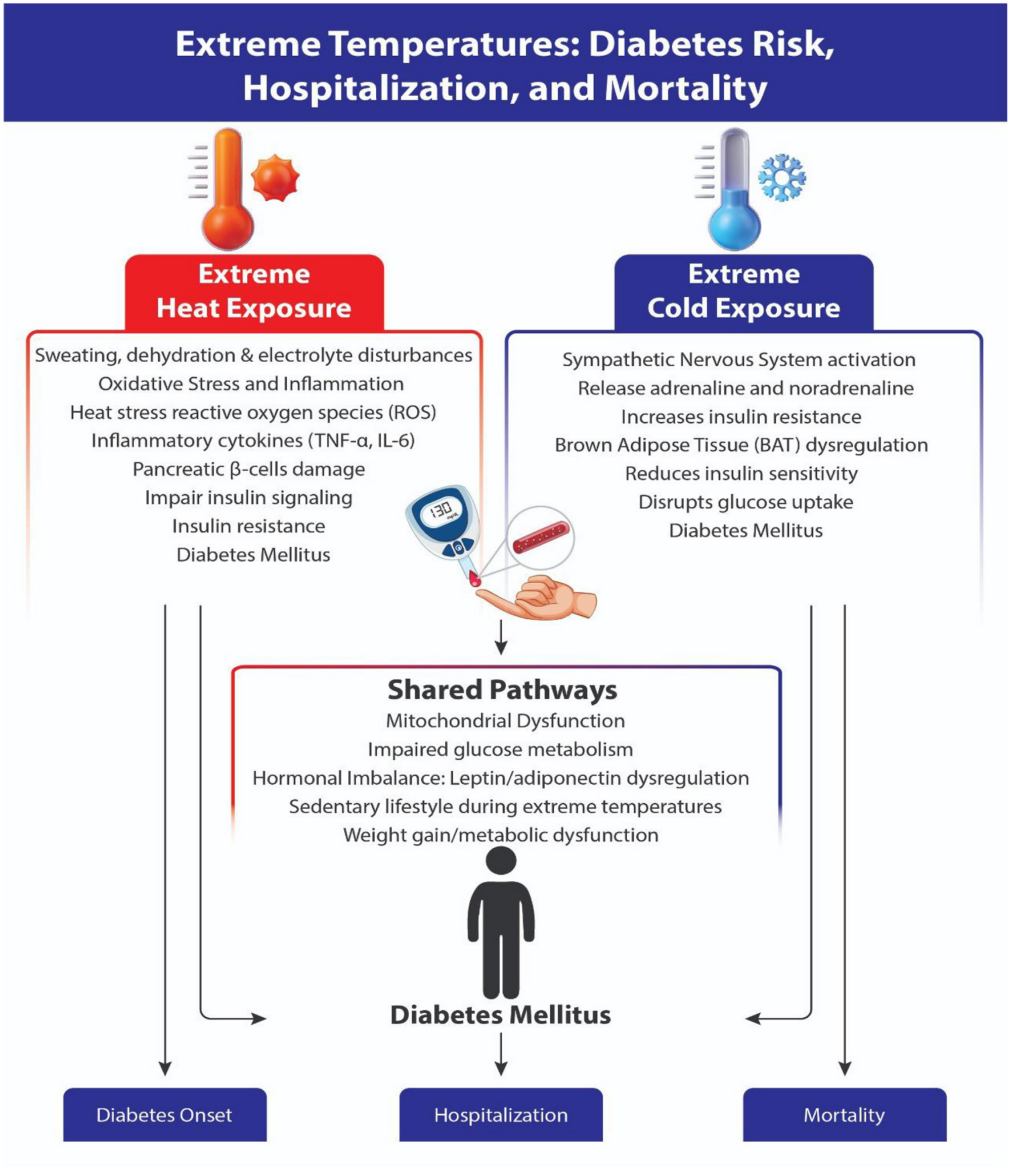
Pathophysiology of the impact of extreme temperatures (heat and cold) on diabetes onset, hospitalization and mortality.

Extreme heat causes increased sweating, fluid and electrolyte loss, dehydration, impaired autonomic nervous system function, and thermoregulatory abnormalities (28). Moreover, extreme heat can cause systemic inflammation (29), insulin resistance and diabetes mellitus (30). However, extreme cold limits people from doing outdoor work, minimizes physical activity, and causes a sedentary lifestyle, which together lead to obesity, insulin resistance, and DM (31). The literature also mentions that extreme temperatures cause mitochondrial dysfunction and insulin resistance (32, 33). All these mechanisms play a role in the occurrence of DM, diabetes-related adverse events, and mortality (Figure 5).

### 4.2 Study strengths and limitations

This study's analysis is based on the combined results from multiple studies across diverse regions and populations, thereby enhancing the generalizability of the findings. The study findings support the identification of a relationship between extreme temperatures and diabetes outcomes. The data provides pooled effect estimates (odds ratios, relative risks) for hospitalizations, emergency visits, or mortality among people with diabetes due to temperature extremes. There are some limitations of our study. First, the analysis is based on the country-level aggregated data and a limited number of studies. Second, because our analyses rely on databases rather than individual patient data, we cannot rule out the possibility of confounding factors. Other limitations include variability in definitions of climate zones, temperature, healthcare systems, and population characteristics, which can affect the consistency of the results. The differences in how outcomes, diabetes risk, hospitalization, ED visits, and mortality are defined or reported can limit comparability. The periods and geographic locations may differ significantly across the included studies, which can affect the applicability of the pooled results to specific contexts. Some studies may not adequately adjust for confounders such as air pollution, socioeconomic status, or comorbidities, which may influence outcomes.

## 5 Conclusions

Extreme weather conditions (heat and cold) significantly increase the incidence of adverse diabetes-related events, hospitalizations, emergency department visits, and diabetes-related mortality. The results underscore the need for future research into the effects of extreme temperatures on glucose metabolism, insulin resistance and the incidence of diabetes mellitus. Reducing the occurrence of climate change and extreme temperatures requires coordinated efforts at all governmental and organizational levels, from global to national, community, and individual. Regional and global health and policymaker authorities must implement strategies to curb ecological pollution and minimize greenhouse gas emissions, protect forests, promote a green environment and improve urban planning, thereby reducing the risk of climate change and diabetes incidence and mortality.

## Data Availability

The raw data supporting the conclusions of this article will be made available by the authors, upon reasonable request.
